# Towards a standardized competency-profile for peer-to-peer-teachers in medical education - a systematic mapping review

**DOI:** 10.1186/s12909-026-09997-9

**Published:** 2026-07-30

**Authors:** Christian Bernhard-Skala, Bernhard Steinweg

**Affiliations:** Medical Faculty of the University of Bonn, Deanery of Studies, Venusberg-Campus 1, 53127 Bonn, Germany

**Keywords:** Peer-to-peer-learning, Student as a teacher, Tutoring, Competency, Qualification, Mapping review, Systematic map, Medical education, Research, Empirical

## Abstract

**Background:**

Medical education research acknowledges “P2P-teaching” as an effective, scalable, and cost-effective possibility for educating students in human medicine. At the same time, the medical field is yet to form an empirically founded and overarching description of necessary P2P-teaching competencies across medical subjects. In order to create an overarching picture of relevant student P2P-teaching competencies in medical education, this article raises the question, “What are the relevant teaching competencies in qualification measures for P2P-teachers in medical education?”.

**Method:**

We performed a systematic mapping review. Our search strategy applied the PICOS-Scheme and the semantic fields “student tutor”, “qualification”, “competency”, “medical” and “empirical” to medical and educational databases, over the period from July to November 2024. A snowballing-system enriched the results, and a selection process according to PRISMA systematically reduced search results from 322 down to 18 peer-review articles for in-depth analysis. This final sample includes peer-reviewed studies from North America, Europe and Australia, drawing from many medical disciplines and published in English and German. For synthesis, we extracted key study characteristics and qualitatively analyzed contents of qualification measures for P2P-teachers.

**Results:**

The analysis brings three medical and 12 student P2P-teaching competencies to the fore. Together these competencies form a competency-profile for student P2P-teachers. The competency-profile shows overlaps with relevant medical teaching frameworks. The relevant studies not only show a weak grounding of P2P-teaching research in learning and teaching theories, but also that they tend to be limited to low levels of evidence.

**Conclusions:**

Our results are limited to the peer-reviewed and English- and German discourse. This specific discourse on P2P-teaching does not show an overarching consensus as to what the focal competencies for student P2P-teachers are, but it shows a remarkable number of implicit and explicit overlaps. Therefore, the P2P-teaching competency-profile for student tutors might serve as a starting point for (and invitation to) an open-discussion across medical schools to create and elaborate on an interrelated evidence-based research strategy on P2P-teacher-training. Against the background of the current state of research methodology, systematically investigating competencies of student P2P-teachers is a fruitful research area.

**Supplementary Information:**

The online version contains supplementary material available at https://doi.org/10.1186/s12909-026-09997-9.

## Background

Peer-to-peer (P2P) teaching has been established and institutionalized in many countries’ medical universities over the last 15 to 20 years [1–4]. Consequently, P2P-teaching formats such as tutoring, peer-mentoring, peer-assisted learning, peer-near learning or students as teachers have become a remarkable phenomenon and a growing part of educational practice in human medicine [5–9]. P2P-teaching takes place across medical subjects and disciplines, especially in anatomy classes and skills labs for medical skills training. The German-Austrian-Swiss Society for Medical Education (GMA) underlines the importance of P2P teaching, among other things, within the framework of the “International Skills Lab Symposium”, a yearly conference organized by its committees “Simulation Persons” and “Practical Skills”, that was established in 2007 [10].

Medical education research acknowledges P2P-teaching as an effective, scalable, and, therefore, cost-effective possibility for educating students in human medicine [4, 11]. In comparison to classical, rather hierarchical learning and teaching processes led by clinical physicians, P2P-teaching is theoretically associated with a high degree of social and cognitive congruence [12]. Therefore, it can lead to high quality and effective learning processes, that are different, but not inferior to their classic counterparts [1, 5, 6, 13–15].

With the growing institutionalization and recognition of P2P-teaching in medical education, a rather small but very important debate has started, on how to systematically qualify students to become good P2P-teachers [4, 16–19]. By that, the medical education discourse acknowledges the focal role of teachers and teaching practices in educational processes, which has been widely emphasized in educational research [20–22]. So far, the debate about how to qualify and professionalize medical P2P-teachers has focused on effective teaching methodologies in terms of formal learning conditions such as time, group size and teaching methods [6, 9, 23]. Furthermore, the debate highlighted content dimensions and the range of necessary P2P-teaching competencies *within* certain medical subjects. Initially, Srinivasan et al. [24] developed competency-profiles for medical teachers. Alongside the Committee for Personnel and Organizational Development in Teaching of the Society for Medical Education, an orientation framework for medical teaching skills of physicians was created [25]. However, an empirically based and comprehensive description of the necessary P2P-teaching skills across medical subjects is still in its infancy. In order to create an overarching picture of relevant P2P-teaching competencies in medical education, the present article raises the question, “What are the relevant teaching competencies in qualification measures for P2P-teachers in medical education?” By answering this question, this article establishes the basis for developing a P2P-teacher-competency-profile that aims to be applicable across human medicine faculties and universities. Raising this specific question, the paper adopts a teacher professionalization approach.

## Method

### Search strategy

A systematic mapping review was conducted [26]. The search was limited to peer-reviewed papers, published between 2014 and 2024. In a preliminary search, limiting the results to 10 years created an amount of results that we could handle effectively. Furthermore, it captured younger medical education developments such as the growing professionalization, the importance of skills labs and the rise of problem-oriented-learning-approaches, which are often associated with P2P-teaching.

The search strategy consisted of a free-text-search in the following databases:

- PubMed
- ERIC
- Fis-Bildung
- PsyIndex

The free-text-search adapted the PICOS-scheme [26] and captured the research question by referring to:

- **P**opulation—as student tutors or student teachers
- **I**ntervention—as training or qualification measures
- **C**omparison—as learning goals of qualification measures in the context of medical education in human medicine
- **O**utcomes—as skills and competencies
- **S**tudy Design—as empirical

Furthermore, we used the asterisk (*) as a wildcard to find variations of the selected search terms.

As the search focused very broadly on teaching competencies of training measures for P2P-teachers in medical schools and universities, the study integrated outcome and intervention into one operator of the search-strategy. The free-text search operationalized the search strategy using German and English search terms presented in Table 1. It was performed over the period from July 2024 to November 2024.

**Table 1:** Search strategy including search terms and Boolean operators

Concepts *Boolean operator*	Search Terms (incl. Boolean operator)
population:**tutor, student as a teacher**	Student* (AND)
	Tutor* (OR)
	Teach*(OR)
	Lehr* (OR)
***AND***	
Intervention and outcome:**competency, qualification, training**	Kompetenz*(OR)
	qualif* (OR)
	skill*(OR)
	competenc* (OR)
	Train* (OR)
***AND***	
Comparison:**within Medical contexts**	medic*(OR)
***AND***	
Study design:**Empirical**	Empir*

As the search strategy focused German and English journal-articles and neglected grey literature as much as literature in other languages, publication bias is probable.

### Selection process

In a first step, the selection process focused on the information provided in title and abstract. Full texts were analyzed in a second step. The following predefined *inclusion and exclusion criteria* applied.

#### Exclusion criteria

- The study concerns educational issues outside the context of medical education in universities or medical schools.
- The study is published in a language other than English or German.
- It is not an empirical study.
- The study concerns the socio-structural characteristics of learning and teaching outside the context of P2P-teaching, but not a training intervention.
- The study examines the teaching competencies of professional lecturers and not those of student P2P-teaching.
- The study deals with the medical competencies of physicians.
- The study is not available via the authors’ host institution.
- The study was published before 2014.

#### Inclusion criteria

- The study is published in a peer-reviewed journal.
- The study examines training or qualification measures of student P2P-teachers in human medicine.
- The study empirically describes the competencies for student P2P-teachers in human medicine that student P2P-teachers need to acquire.

**Fig. 1:**
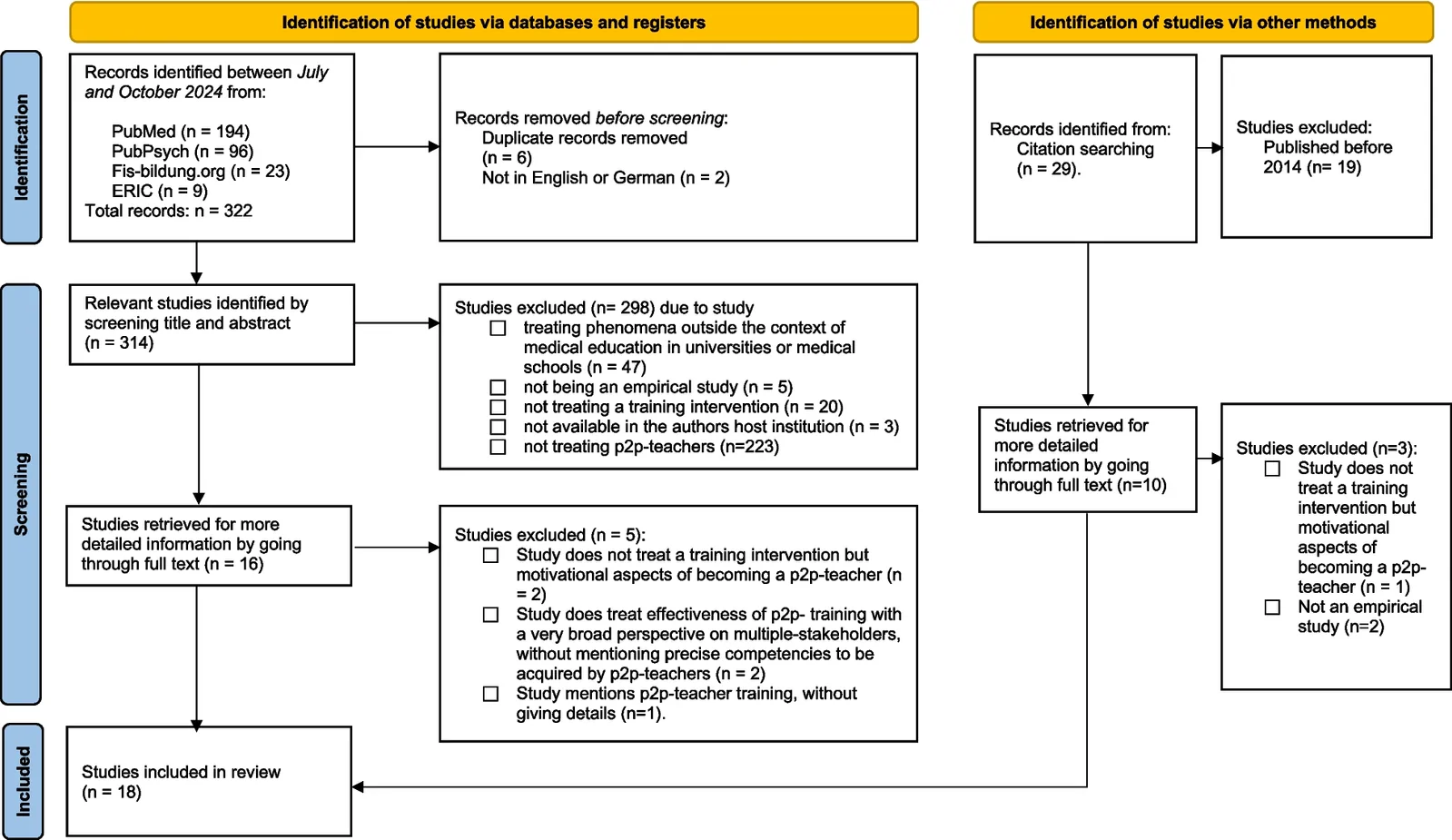
Selection process according to PRISMA

Figure 1 presents the selection process according to PRISMA.

### Data extraction, appraisal and synthesis

A mapping review “maps out and categorises existing literature from which to commission further reviews and/or primary research by identifying gaps in research literature” [26]. Therefore, when appraising full-texts, the PICOS-scheme was applied in a slightly adapted manner: P2P-teachers being the participants, a training program with explicit learning goals being the intervention, with the competencies and learning goals of the trainings being the comparator and the outcomes. Our focus was on study designs, in order to identify methodological gaps. As a result of the in-depth analysis, 11 more articles were excluded. So, a final 18 articles formed the focus of the analysis. The initial idea to identify evidence of effective training methodologies was abandoned because the studies’ evidence level was rather low. Generally, groups of peer-teachers in a skills lab or other teaching entity are rather small. Therefore, samples do not normally exceed more than 20 participants per training measure. Furthermore, the learning goals of the qualification measures were very diverse. Annex 1 presents the details of the synthesis of the 18 full texts.

With our decision, not to focus on training effectiveness, but rather on goals and contents of qualification measures, we decided not to perform assessments of study risk of bias, but chose to develop categories inductively within a Qualitative Content Analysis approach [48]. The inductive coding process followed heuristically common steps of planning and creating a train-the trainer course. Selection, screening, data extraction, and coding were conducted by one author (C-BS), all aspects were intensively and critically discussed between the two authors.

## Results

Using the methodology described above, the authors identified and analyzed 18 empirical studies on P2P-teaching skills. These studies focus on data from North America (USA [23, 27, 31] and Canada [32]), Europe (Germany [8, 11, 12, 33–35], Netherlands [36] Greece [6]), and Australia [7]. Furthermore, two reviews with an international perspective were part of the sample [4, 37]. The descriptions of P2P-teacher-training, we found, come from multiple medical specialties and subjects – general and internal medicine [11, 33, 35], neuroanatomy [6], respiratory pathophysiology [29], and also from across disciplines, institutes and professions [4, 7, 8, 12, 23, 27, 28, 30, 32, 34, 36, 37]. The studies date from 2014 to 2022, with peaks in 2022 [4, 8, 34, 36] and 2020 [6, 12, 23, 27, 30]. No studies from 2018 met criteria. The majority of studies focusses cross-year-teaching [6, 11, 23, 27–31, 33, 36], while none of the studies explicitly focusses on same-year-teaching. The other studies [4, 7, 8, 12, 32, 34, 35, 37] do not provide an information whether they choose a cross- or same-year-teaching approach or integrate both approaches.

### Theoretical approaches

The empirical studies mainly report empirical findings; they approach teaching and learning via learning goal-orientation and learning success. Quite a number of articles refer to social and cognitive congruence, only two of them use corresponding theoretical approaches [12, 34]. The few references to learning or teaching theory refer to theoretical stress-models [33], experience-based learning theory [7, 36], attribution approach [6], organizational, pedagogical and affective support [7], the feedback model of Van de Ridder [27] and, finally, Vygotsky’s zone of proximal development [28, 32]. Generally, the role of theory is rather weak, and the foundation of the studies in (learning) theories or theoretical models is limited.

### Research designs

The designs in the sample encompass two reviews – one scoping [37] and one narrative [4] – and 16 empirical studies – six explorative studies [8, 11, 12, 33, 34, 36] as well as 11 evaluation studies [6, 7, 14, 23, 27, 32, 34]. Ten single case studies dominate the sample. Their data are limited on one P2P-programme in one medical school or university at one point of time [7, 11, 23, 28, 34]. The remaining studies encompass four longitudinal case studies [6, 27, 34, 36] and one test-development [12]. There is only one cross-organizational survey [8]. Ten studies choose a quantitative approach [6, 8, 12, 23, 27, 28, 30, 34, 35] – mainly survey-methods, sometimes with additional open questions [23, 33, 35] – five studies follow a mixed-method approach [28, 30, 32, 33]. Two publications report a classic qualitative (focus group) design [11, 36]. The designs of the sample twice [28, 29] exceed Kirkpatrick’s levels of evaluation [38] satisfaction (level 1) and learning (level 2). Studies in the sample approach level 2, mainly via subjective self-assessment by the participants. In more than half of the empirical studies [11, 23, 29, 32, 34, 36] the number of tutors involved is lower than *n* = 20. Two studies [6, 8] do not even involve student tutors in their design at all, but instead focus on organizational structures and student learning. In the remaining five empirical studies [7, 12, 27, 28, 33], the number of tutors ranges between *n* = 48 [28] and *n* = 149 [12].

### Content: What teaching competencies are relevant in qualification measures for P2P-teachers?

As a first result in terms of teaching competencies, the studies show that the practices of qualifying P2P-teachers, either explicitly or implicitly integrate medical skills with teaching skills. Sometimes, medical subject content such as anatomy [8] or internal medicine [11] is explicitly part of the P2P-training program; sometimes the teaching skills implicitly require elevated knowledge of medical subject content (e.g. providing feedback on medical performance [11, 27]). Following the data from the analysis, medical subject content also includes the technical skills of handling technical equipment (such as ultrasound devices) [33], as well as interprofessional content [7]. Here, the data focus, for example, on structured patient handover according to ISBAR.

Beyond these medical contents and skills, the analysis revealed 12 general medical education and training competencies of great interest for practical programs for P2P-teachers and of the ongoing academic P2P-teacher debate. Table 2 provides an overview of all the competencies we defined, and furthermore includes anchor examples and quotations from the studies.

**Table 2:** Overview of P2P-teaching competencies, including anchors and mentions in the data

Category	Teaching competency/competencies	Anchors	Mentions
*Medical contents*			
**Medical contents**	Medical Knowledge and Skills	• “Internal medicine” • “Anatomy”	[8, 11, 30, 33–35]
	Dealing with technical equipment	• “Ultrasound”	[33]
	Interprofessional contents	• “ISBAR”	[7, 8]
*(Medical) Educational and Training Knowledge & Skills*			
**Creation of an instructional design**	Application of learning theories	• “Formulate guiding principles on which effective teaching can be built” • “Empathy” • “Interest in students as learners” • “Respectful behavior towards difficult participants”	[4, 6–8, 27, 28, 30, 32, 34, 35]
	Course planning and course preparation	• Course planning, curriculum development	[7, 8, 29, 30, 33]
	Coordination within the organizational framework of a medical school:	• Show professionalism by fulfilling expectations of section leader via attendance, dress, timely submission of marks to course director • “The role of PT” • teamwork • collaboration • planning and division of labor	[6, 28, 33, 36]
	Application of teaching methodologies	• Peyton’s four steps • Education through simulation • Questioning skills • methods to facilitate laboratory sessions, teaching techniques for adult learners • clinical and procedural teaching • conducting medical exercises	[6, 7, 11, 14, 23, 28, 30, 33, 35]
**Instructional delivery**	Giving presentation	• Presentation	[32]
	Creation of a learning environment	• “Situational awareness” • “Getting to know and engaging the learner”	[23, 29, 31, 34]
	Leadership in (small) groups	• “Small group teaching” • “Group instruction” • “Leadership role demands”	[4, 7, 8, 23, 28, 29, 32, 33, 35]
	Time management	• Time pressure	[29, 33, 34]
	Learning assessment and feedback provision	• Pendleton’s feedback model • DeRidder Feedback-Model • Giving and Receiving Feedback	[4, 7, 8, 11, 23, 27–30, 34, 37]
**Professional development**	Evaluation of learning and teaching processes	• Distinguish between, and apply, formative feedback and summative assessment	[4, 8, 28]
	Self-reflection	• Show reflective ability regarding their own teaching • Perception of peer-teaching performance	[8, 28, 30]
**Organizational competencies**	Organization of large-scale educational events	Learning objectives 1. organize a large-scale educational event in a team 2. fulfill a specific role in the team and take final responsibility 3. insights in additional matters such as budget/finances, (fire) safety, logistic support and public relations 4. manage peers and students 5. clear and concise communication with all parties involved and establishment of contacts with external partners 6. systematic work 7. promote good cooperation within the team	[36]

Working with these competencies will require more than naming categories and anchors, but also a profound reflection of definitions and of learning goals. Therefore, the following detailed description of how the competencies are rooted in the data will help when setting up future training and research designs.

- Application of learning theories: The data show that quite a number of P2P-teacher-trainings [4, 6–8, 28, 30, 32] explicitly treat “learning theories” [30] and “educational principles” [7] in terms of “guiding principles, on which effective teaching can be built” [28]. Unfortunately, the data only provide scarce information as to what these theories are and to what degree they represent paradigms or theorems or even scientific theory. The data only infrequently point out “clinical reasoning” [30] and the very broad term “principles of learning theory” [4, 32]. Furthermore, the data refer to specific behaviors such as “interest in students as learners” [34] or “respectful behavior towards difficult participants” [35]. We categorized these as an application of learning theories, as they do indicate normative assumptions about good learning and good teaching, and represent artifacts of “epistemic beliefs” [39] and “subjective learning theories” [40]. Reflecting epistemic beliefs and subjective learning theories are an important part of teaching professionalization.
- Course planning and course preparation: The data bring up the subject of planning and “preparation” [29, 33] in many ways. Requirements such as “lesson planning” [23], “planning and delivery” [7], “realistic planning” [36], or “content of educational planning” [8] show a need for competencies that allow P2P-teachers to systematically develop a blueprint for their teaching before crossing the threshold of a course room and meeting a group of peer-students.
- Coordination within the organizational framework of a medical school or university: The data show a whole range of issues of coordination within an organization, when it comes to teamwork or to coordination with faculty and/or technical staff [6, 28, 33, 36]. Generally, the data mention issues of coordinating team teaching in P2P-classes or coordination with a supervisor [6, 33, 36]. Erlich and colleagues mention the strongest indicator for P2P-teachers having to integrate into an organization: P2P-teachers should “[s]how professionalism by fulfilling expectations of section leader via attendance, dress, timely submission of marks to course director” [28].
- Application of teaching methodologies: Although the application of teaching methodologies is present in the data [6, 7, 11, 14, 28] the studies make rather vague references to specific teaching methodologies. Sometimes they point out concrete methods such as Peyton’s four-step method [7] or simulation [30]. In addition, “questioning skills” [30] are pointed out.
- Giving presentations: Giving presentations is a classic teaching skill in higher education. In the P2P-teaching data, presentation skills appear only once [32] as being important. This indicates another specific about P2P-teaching: since P2P-groups tend to be small, giving presentation might be considered a teaching skill for large groups. Therefore, presentation skills might not receive a lot of attention whether in the discourse or in the field.
- Creation of a learning environment: The data refer to the active creation of a “conducive” [31] or “an open and non-judgmental” [34] learning environment in terms of high social and cognitive congruence in P2P-learning-processes. The medical education discourse claims social and cognitive congruence to be a characteristic of P2P-courses that helps students to ask questions and to reflect openly when dealing with insecurities. In the context of the present article, “situational awareness” [29] as well as “getting to know and involving the learner” [29] are categorized as being integral parts of creating a positive and open learning environment.
- Leadership in (small) groups: A number of studies [4, 7, 8, 23, 30, 32, 33, 35] highlight group dynamics and leading (small) groups as an important aspect. Small group-arrangements are a characteristic of P2P-courses. The data refer to skills such as facilitating student centered group discussion [28], “group management” [8], or “group instruction” [33]. All these activities relate to small groups and imply a certain degree of leadership skills and taking a leadership role in the learning process of peers. At the same time, one of the studies in the sample refers to taking a leadership role as a major stressor for P2P-teachers [33]. This underlines the importance of training in this field.
- Time management: The data highlight time management as both an important skill [29] and as a major stressor [33], while “time to answer student questions” appears as a major factor when building up social coherence [34]. Time management differs from course planning. While course planning happens before the course, time management is a skill which P2P-teachers need to perform during the course.
- Learning assessment and feedback provision: Facilitating clinical skills acquisition through formative assessment and feedback represents an essential skill for P2P-teachers and is mentioned numerous times [4, 7, 8, 11, 23, 27, 30, 34]. The study of Stenberg and colleagues [37] provides important insights for its use in higher healthcare education. The present data relate to different models of feedback provision to peer-students (e.g. Pendleton [7] or Van de Ridder [27]). It is remarkable that the data refer to giving feedback more frequently than to receiving feedback. The present category highlights feedback provision. Receiving feedback for own teaching activities, however, is part of evaluation skills (see below).
- Evaluation of teaching processes: Although evaluation closely interlinks with assessment and feedback in the data, we chose to elaborate on evaluation as a separate skill. The data of the sample distinguish between summative and formative evaluation of teaching processes [4, 7, 28], which implies a process of judging the quality of one’s own teaching and not only measuring the learning progress of others. The data do not indicate if evaluation is linked to any kind of standardized processes such as surveys or open feedback to the P2P-teacher. Evaluation skills, in this narrow sense of systematically judging one’s own teaching, appear three times as relevant for P2P-teachers, in our data.
- Self-reflection: Self-reflection is an up-to-date skill in the data. It is mentioned three times [8, 28, 30], although without defining it even vaguely.
- Organization of large-scale educational events: Organizing large-scale events is probably not a day-to-day activity of P2P-teachers but might be a skill or a task that becomes relevant once a year or a semester. In the data, this skill appears only in one study [36] and reflects a whole range of necessary project management skills for organizing a large-scale educational event. In the data, the event exceeded 1000 participants. At the same time, large-scale is a subjective term, and can indicate a weekend course of 20 persons. In this particular case, large-scale refers to the fact that local regulations normally limit the size of curricular medical study groups that elaborate on practical skills to six participants.

## Discussion

The results show quite some overlaps between different approaches for how to qualify P2P-teachers, but do not reach a consensus. The overlaps consist for example in competencies such as the application of learning theories, learning assessment and feedback provision, small group leadership, and, the application of teaching methodologies. These competencies appear nine to eleven time as learning goals or contents of P2P-qualification measures. Giving presentation and organizing large-scale educational events appear to be rather exotic: In our material, we found the latter ones only once. Surprisingly, digital competencies are not all present in the data and form a gap.

**Fig. 2:**
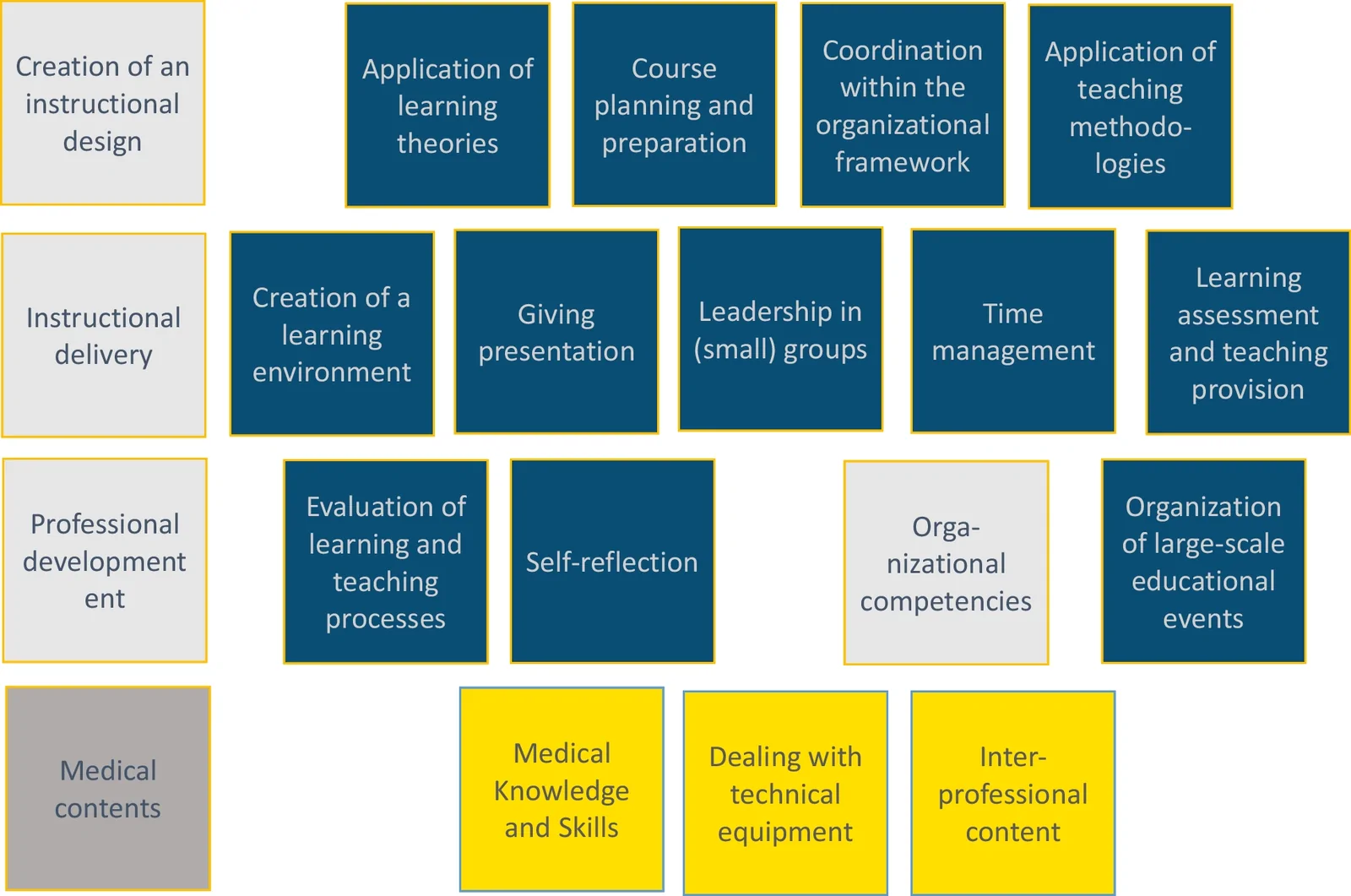
Draft of a qualitative competency-profile for qualifying P2P-teachers aggregated from 18 studies

We integrated the results into a first draft of competency-profiles for student P2P-teachers in human medicine (see Fig. 2). The draft reflects the interplay of medical skills, on the one hand (yellow colors), and teaching skills (blue colors), on the other hand: Although medical skills and teaching skills are separate from each other, they represent two sides of the same coin: In the context of medical P2P-teaching, one does not work without the other. We have integrated the different medical and teaching skills as subcategories into the profile without judging them as more important or less important. As this draft of a P2P-teacher competency-profile should serve as a starting point for further discussion, the figure does *not* represent quantities or even training priorities, but opens up the qualitative variety and the range of the competencies that play a role when qualifying P2P-teachers.

Comparing both the teaching skills of the Canadian CanMEDS Framework [41] and the German National Competence-Based Learning Objectives Catalogue [42], the student P2P-competency-profile presented above proves compatible.

The widely recognized Canadian physician competency framework CanMEDS explicitly outlines six competencies within the physicians’ role as teacher. Four of these six competencies are explicitly part of our student P2P-competency-profile: “promote a safe learning environment”, “plan and deliver a learning activity”, “provide feedback to enhance learning and performance” and “assess and evaluate learners, teachers and programs in an educationally appropriate manner”. Our P2P-competency-profile integrates this last aspect in a more detailed manner by delineating the difference between evaluating learning and teaching activities. Furthermore, the CanMEDS role classifies role-modeling and ensuring patient safety to be within teaching activities. Role Modeling might be considered a part of the self-reflection competencies of P2P-teachers. Moreover, it is an open question if patient safety might be an issue for student P2P-teaching. Indeed, each medical school and university needs to answer this in their specific P2P-context – e.g. looking at P2P-blood draw-courses, where participants regularly claim to practice cannulation with a special skills trainer or with each other.

Compared to the role of the teacher from the German National Competence-based Learning Objectives Catalogue (NKLM) [42], again, the P2P-competency-profile shows a number of overlaps and proves compatible. The role of “the scholar” encompasses selection, evaluation and application of content, teaching and examination strategies, as well as giving feedback and reflecting on teaching–learning situations. So, five competencies from our P2P-teaching-competency-profile are clearly named in the NKLM. Another open question that needs to be discussed further in the P2P-specific context of a medical university: Whether the *presentation of scientific studies* defined in the NKLM-Framework has the same meaning as *giving a presentation* as defined in our P2P-profile.

So, overall, the teaching competencies in our student P2P-competency-profile are mainly in line with relevant frameworks for professional education of physicians, even though they apply to teaching peer-students.

It is worth noting that although the NKLM-framework places the acquisition of teaching skills in the late phases of medical education (e.g. second clinical stage of study or clinical year), even first semester students act well in P2P-teaching situations as anatomy tutors or mentors. Therefore, one might agree with calls for the early integration and longitudinal implementation of P2P-programs and medical education skills into human medicine curricula [4]. Peters and colleagues [43] provide a portfolio of classroom and workplace-based teaching activities that might prove helpful in terms of entrustable professional teaching activities.

From an educational point of view and against the background of various competency-frameworks for school and adult teachers [44, 46], it is astonishing that digital competencies are not reflected at all in the medical P2P-teacher literature. In future, it will be important to consider digital competencies when qualifying P2P-teacher for blended learning, XR, AR or AI application. Again, compared to educational competency-framework, our draft reflects the importance of cooperation and coordination with other teaching staff, which represents an important prerequisite for innovation of training. Generally, the profile is in line with main categories such as professional development, knowing and applying learning theories and teaching methodologies and mastering teaching contents.

From a research point of view, it will be important to elaborate on all the competencies of the profile and to ground them in theory. For example, it is unclear what the most important learning theories and principles to be taught to P2P-teachers are. Further, the creation of a sound learning environment seems to be limited to social aspects, although spatial, digital and security aspects have an important practical and theoretical role to play. Elaborating the theoretical backgrounds of competencies in order to create a sound competency-framework for P2P-teachers will be a large task. This cannot be accomplished by any one medical university, but will require discussions, negotiation and empirical investigations across medical schools and universities.

Furthermore, the findings again confirm [4, 9, 28] that medical educational research on P2P-teaching remains on a rather low level of evidence. A large part of the studies evaluates learning effects on the level of satisfaction and the self-assessment of learning effects. Likewise, the majority of studies works with self-developed surveys, that do not possess validity beyond their own institution [47]. Finally, the empirical basis of the studies remains small in terms of cases involved. In about the half of the studies, n is lower than 20. This is probably due to the structural fact, that one institute or one skills lab does not normally exceed 20 P2P-teachers. The studies which rely on a high number of cases are longitudinal ones. So, there is a need for more cross-institutional and multi-center studies in the field of P2P-teacher research. This will not only increase the empirical basis of P2P-research in terms of numbers and the validity of results across organizational contexts, but also the impact of P2P-arrangements. The proposed draft of a competency-profile might be a first step to set up cross-institutional designs, in the long-run: Medical education researchers might align their P2P-research with the competency-profile, set up theoretical foundations for the competencies, develop and validate tests and survey items, and share them in publications. That way, medical schools and universities might use and adapt these results for their research within their own institution. In short: This draft of competency-profile might integrate and validate existing and future research in terms of empirical basis and theoretical foundation, and also in applicability in other institutional contexts.

Similarly, from a practical medical education point of view, this competency-profile might serve as a draft when developing, implementing and standardizing medical education curricula for P2P-teachers. Particularly – and this is important to underline – since the competency-profile in itself does not represent a curriculum. It serves as a starting point to developing learning goals and programs. Skills labs, clinics, and institutes that are engaged in P2P-teaching can focus competencies which represent the roles of P2P-teachers in their respective medical education programs and start with training measures. Or they can leave competencies aside, if they judge them unimportant. Finally, the competency-profile is far from complete. So, if curriculum developers (or other persons in charge of P2P-teachers) find that certain competencies are lacking (e.g. digital competencies), they are free to add them. In brief: The competency-profile presented in this paper represents a contribution and an invitation to a cross-institutional discussion of medical education practitioners and researchers.

### Limitations

The competency-profile presented above represents a snapshot of the scientific medical education discourse that takes place in peer-review-journals. Publication bias is likely, since successful or innovative P2P programs are more likely to be published than unsuccessful ones. Furthermore, our snapshot focusses strongly cross-year-P2P-teaching and appears to neglect same-year-P2P-teaching. Although we have identified a broad range of competencies that form a starting point for discussion, we do not know the extent to which praxis has already formed a consensus. In other words: In our model, we can only describe P2P-teacher-trainings that are published in journals. We need to be conscious that there are numerous high-quality and valid P2P-teacher-qualification measures that have not yet shared their approaches or results in journals. So, we may have missed results that are hard to find. Furthermore, our search strategy did not include the term “peer”, which is probably a keyword in the discourse. Finally, during the research process, we decided to leave aside the levels of evidence of P2P-teacher-training, because we found the evidence to be rather low. The levels of evidence might be worth an in-depth analysis, although the empirical basis will probably be rather small.

## Conclusion

At present, there is not an overarching consensus on what the relevant skills for teaching student P2P-classes are, or should ideally be. Happily, the discussion shows a number of overlaps, and this can serve as a starting-point for a common discussion and innovation. Twelve teaching competencies and three broad categories of medical competencies form a first draft of a competency-profile for P2P-teachers, which is mainly in line with relevant medical qualification frameworks for professional medical teachers. Therefore, the competency-model might serve as an evidence-informed starting point for the discussion of P2P-training measures and professionalization of student P2P-teachers within the medical education community. Furthermore, our analysis shows that researching P2P-teacher-training is a fruitful activity: There is not only an empirical basis to be improved in terms of methodology, but also an important field of practice that shows significant practical and scientific potential.

## Supplementary Information

Supplementary Material 1.

## Data Availability

No datasets were generated or analysed during the current study.

## Abbreviations

GMA: "Gesellschaft für Medizinische Ausbildung “ – German-Austrian-Swiss Society for Medical Education (GMA)
NKLM: “Nationaler Kompetenz- und Lernzielkatalog Medizin” – German National Competence-based Learning Objectives Catalogue (NKLM)
P2P: Peer-to-peer
