# Supplementary material for: Towards a standardized competency-profile for peer-to-peer-teachers in medical education - a systematic mapping review
Source: BMC Med Educ. 2026 Jul 30;26:1230. doi: 10.1186/s12909-026-09997-9 (PMC13430701; doi:10.1186/s12909-026-09997-9)
Supplement: Supplementary file 1 — Supplementary Material 1. [file 12909_2026_9997_MOESM1_ESM.docx]

| **Author**  *Geographical and medical context* | **Approach**  *Teaching competencies and learning goals of qualification measure* | **Theoretical background** | **Design**  Method  *Sample population* | **Results** | **Strengths and Limitations**  *remarks* |
| --- | --- | --- | --- | --- | --- |
| **Bockrath et al. [27]**  *Northwestern University Feinberg School of Medicine, Chicago USA*  *History taking, communication, and physical exam skills M1/M2 small groups.*  *cross a number of medical disciplines* | Cross-year teaching  **Alignment and evaluation of two existing “Residents as teachers” and “Medical students as teachers” programs**  *giving effective feedback as a clinical teacher; assessing and improving personal performance through the creation of an individual improvement plan.* | **Feedback-Model of Van de Ridder** | **Longitudinal study (2013-2016)**  Survey and document Analysis of written feedback  *data from residents and M4s (2011 to 2016)*  *n=79 residents, n= 445 M4students* | The program quality rises in terms of satisfaction over time. | selection effects due to voluntary participation of residents  no cross-organizational design |
| **Burgess et al. [7]**  *Sydney Australia*  *interprofessional: Medicine, Pharmacy and Health Sciences* | No information, whether cross-year or same-year teaching  **Development of a peer-teacher-training for interprofessional teaching**  *Module 1: Introduction to the program*  *Module 2: Planning and Delivery*   - *understand and assess educational principles* - *planning activities* - *define learning outcomes, teaching methods and assessment methods*   *Module 3: Teaching a skill and feedback provision (Peyton’s four steps, Pendelton’s feedback model)*   - *assessment of learning achievement*   *Module 4: Assessment and Feedback provision*  *Module 5: Small group teaching*  *Module 6: Effective clinical handover (ISBAR)* | **Experience based learning theory**  **organizational, pedagogic, affective support** | **Single case study**  Pre-post-design qualitative and quantitative survey  *115 attended & 90 completed the program*  *N=80 completed the questionnaire* | The program seems to be effective in terms of Learning outcomes  Creation of a professional mindset towards teaching | High Dropout  Voluntary participation might lead to a bias. |
| **Cohen et al. [4]**  *international* | Cross-year and same-year teaching  **Narrative Literature review**  *Medical content*  *small-group facilitation, principles of adult learning, and providing effective feedback and evaluation* | **/** | **Narrative review, limited to scholar articles,** ranking of studies along with Kirkpatrick’s levels of evaluation | While medical students often engage in teaching roles, formal teaching skills training is less frequently provided.  Most of the trainings target senior medical students and are elective courses.  About one-fifth of the curricula are delivered longitudinally.  Various benefits: positive student reaction, enhanced self-reported confidence and preparedness for future teaching roles, and increased interest in pursuing an academic career. | Systematic work |
| **Erlich & Shaughnessy [28]**  *USA*  *Final year students (12-week curriculum, weekly sessions) teaching first year students in “Medical Interviewing and the Doctor–Patient Relationship”* | Cross-year teaching  ***Evaluation of the Student-teacher education program (STEP)***  *(1) Formulate guiding principles on which effective teaching can be built; (2) Apply a variety of methods to teach during a patient interview; (3) Distinguish between, and apply, formative feedback and summative assessment; (4) Deliver feedback using various methods; (5) Facilitate an effective student-centered group discussion; (6) Observe and assess students conducting a patient interview; (7) Solicit feedback on their own teaching;  (8) Assess student written assignments;  (9) Demonstrate qualities of mentorship for first-year students;  (10) Teach with confidence;  (11) Show reflective ability regarding their own teaching;  (12) Show professionalism by fulfilling expectations of section leader via attendance, dress, timely submission of marks to course director.* | **Zone of proximal development (Vygotsky)** | **Single case study**  Kirkpatrick’s four levels of evaluation  Mixed methods: pre-post-questionnaires, ethnographic field observation, analysis of formative and summative numeric teacher assessments, Test-results in OSCE  *N=48 Tutors (13 intervention group/35 Control group)* | Significant differences in focus skills: mentoring, feedback, dealing with difficult learners.  The training creates a positive attitude towards teaching.  The interlinked training approach of theory and practice is new.  Too few studies evaluate students as teachers beyond satisfaction and subjective assessment of learning achievement. | In comparison to other studies in the field, it is a methodologically very strong study.  The study is limited to one university context. |
| **Gottlieb, Epstein & Richards [29]**  *Harvard Medical School, USA*  *M4s to co-teach small groups of M2s together with faculty members*  *Respiratory Pathophysiology* | Cross-year teaching  **Evaluation of a M4 co-teaching program**   - *Pre-course “Teaching to Teach” contents* - *Preparation,* - *Situational awareness* - *Getting to know and engaging the learner* - *Facilitating discussion* - *Giving and receiving feedback*   *Course was led by the course director* | / | **Single case study**  *Mixed-method Surveys*  *N = 114 Learners N=13 student co-teachers N=13 faculty members Informal measure: reflection essays of M4*s | MS2 perceptions   - 90% of tutors increased their understanding. - Tutors are well prepared. - 92% tutors teaching is as good or better than faculty members.   MS4s   - 92% enjoyable experience. - 100% prepared for future teaching, would participate again. - Struggle with time. - Thinking on the spot. - Balancing roles - Knowledge/material - enthusiasm | Kirkpatrick level 2 and 3  Multiple perspectives  Not known how many students in all took part in the class  *One time evaluation without comparable data.* |
| **Harvey et al. [30]**  *Uniformed Services University, USA* | Cross-year teaching  **Evaluation of M4s teaching M1s**  *Learning Theories*  *Formative Assessment: How to Check for Understanding*  *Resident as Teacher: Questioning Skills*  *How to Conduct Small Group Learning*  *Curriculum Development*  *Education through Simulation*  *Clinical Reasoning*  *Introduction to Clinical Skills*  *Reflective Practice*  *+ medical contents* | / | **Single case study**  **Mixed method**  (1) Verbal feedback collected at mid and final feedback exchange sessions  (2) Underclass student surveys  (3) MS4 course evaluation surveys  (4) MS4 activity log.  Focusing satisfaction and subjective achievement of learning goals of M4s, as much as satisfaction with teaching processes of M1s  *M4s n=12*  *M1s n=59* | Very high satisfaction of M1s and M4s  M4s show high satisfaction especially with methodology employed | Positive bias due to M4s created the course themselves. |
| **Herinek et al. [8]**  *Institutes and schools across Germany*  *Interprofessional learning, no focus on certain medical specialties* | No information whether same-year or cross-year teaching  **Describing contents of qualification measures for peer-tutors*.***   - *Basics of learning/education theory* - *Content of educational planning* - *Group management* - *Teaching, assessment and evaluation* - *Communication/feedback* - *Reflection/self-reflection* - *Subject content (e.g. anatomy)* | / | **Exploratory quantitative study** Online-Survey (own development)  *medical education providers (physiotherapy, nursery and other medical schools and medical universities) across Germany*  *Total n=100 / n=18 medical universities* | 46 providers use PAL  32 providers use monoprofessional PAL courses/ 6 schools in human medicine use monoprofessional PAL courses  12 providers use interprofessional PAL n= 12/ 3 medical universities use interprofessional PAL   - All dimensions of teacher training were relevant. None was added. - few differences in training for peer-teaching in monoprofessional and interprofessional contexts seem to appear - Subject content in practice is a relatively important part of PAL-Training. | - Snapshot across medical education providers giving a German picture representing 50% of German medical schools. - Sample size is too small, to draw conclusions about general differences between. The sample size is very small in interprofessional training (n=9). |
| **Hundertmark et al. [33]**  *University of Heidelberg, Germany*  *Institute of General Internal and Psychometric Medicine*  *p2p-courses (Aalplus - Anatomie am LebendenPlus & Abdominale Sonographie)* | Cross-year teaching  **Measuring general and p2p-specific stressors of p2p-Students**  *General stressors:*   - *Studies and exams* - *Teaching and preparation* - *Doctorate* - *Part-time-jobs* - *Travel, family visits and Erasmus-stays* - *Housing search and moving* - *illness (own and family members)* - *social engagement* - *other or unspecific*   *p2p-specific stressors – during sessions:*   - *Group characteristics* - *Team teaching coordination* - *Organizational conditions* - *Leadership role demands* - *Time pressure* - *Group instruction* - *Uncertainties in medical knowledge* - *Supervision* - *Participant characteristics* - *Personal discomfort due to illness or hunger* - *Ultrasound difficulties in terms of the investigated body* - *poor Participant performance* | **Stress-theory** | **Single case study**  Mixed-method-Design:   - Collection of psychological (subjective stress, affective state) and physiological data (Cortisol, heartrate) by use of VAS-Stress and PANA - Additional open question treating every day and p2p-specific stressors   *N=60*   - *36 AaLplus and tutors 24 sonography tutors* | Generally, p2p-teachers successfully cope with stress and quickly recover after giving lessons. The stress level is low and, therefore, has positive effects on p2p-teaching. | - standardized comparative study across courses, using validated instruments - sample is relatively big - self-selection due to voluntary participation is not excluded - medical faculties’ organizational structures and fields of application for peer teaching are diverse, which is why other tutor programs come with potentially different requirements and demands on tutors that may result in different stressors and stress profiles - training measures for tutors are not mentioned although they exist. |
| **Karamoudis et al. [6]**  *Greece*  *Neuroanatomy, 2nd year course* | Cross-year teaching  **Introduction of a peer-teaching program in one course**  *Perception of peer-teaching performance*  *the role of the PT,*  *learning theory,*  *teaching skills,*  *teamwork,*  *methods to facilitate laboratory sessions,*  *course learning objectives* | **Attribution approach** | **Longitudinal retrospective survey**  *N=527 students*  *M2 – M6* | Attributes of PTs related to   1. the student’s opinion of the importance of laboratory activities 2. final grades 3. amount of training that PTs | Limitations:   - Limited to laboratory tutoring - Voluntary participation - Co-occurrence does not mean causality between final grades and perception of PT) |
| **Kobayashi et al. [23]**  *USA, University of UTA*  *4 year SaT-Program* | Cross-year teaching  **Evaluation of a four-year SAT-Program**  *feedback, lesson planning, teaching techniques for adult learners, learning environment, small-group teaching, clinical and procedural teaching techniques, delivery of morning report-style sessions*  *+ capstone project* | */* | **Single case study**  retrospective pre/post electronic survey  7-point Likert-scale Items +  Open-ended questions  N=18 | increased confidence in teaching peers,  improved ability to give and receive feedback,  increased awareness of strengths and weaknesses as educators.  live teaching practice with timely feedback | Short and brief |
| **Krautter et al. [11]**  *Heidelberg, Germany*  *Internal medicine*  *On-Ward*  *3^rd^ year students (6^th^ semester)* | Cross year-teaching  **Support of rotation and workbased learning in the ward**  *The intervention included specific content of internal medicine, feedback training, and didactic methods to apply as a PAL tutor.*  *6-hour Peer-Assisted-Learning (PAL) course*  *+ Manual*  *PAL can only be regarded as PAL, when tutors are trained.* | / | **Single case study**  Qualitative approach with focus groups  *Students:*  *N= 168*  *IG = 88*  *CG=88*  *7 focus groups with 6 participants each*   - *4 focus groups within IG* - *3 focus groups within CG*   *content analysis*  *Tutors:*   - *N=14 final year students (5^th^ year)* | Tutors   - motivate students, - help them to integrate into the ward team, - provide a non-fear-based working relationship whereby students’ anxiety regarding working on ward decreases.   The control group   - had to rely on autodidactic learning strategies when neither supervising physicians nor final-year students were available.   Students in the intervention group had less contact with physician.  PAL is valuable, but cannot a substitute experienced physicians | - Focusses on work-based learning - The results of the analysis are not drawn across groups, but only within groups. This leads to rather description than analytic results. |
| **Loda et al. [12]**  *University of Tübingen, Germany*  *various institutes of human medicine such as medical history, anatomy, internal medicine, physiology, skills lab and surgery* | No information whether same-year or cross-year teaching  **Development of test-items**  *See Loda et al [34]* | **social and cognitive congruence as a decisive factor in p2p-teaching** | **Longitudinal approach**  Quantitative approach  *Sample*:  *N=527 students*  *N=149 student tutors* | Instrument for testing cognitive and social congruence between students and student tutors,  Creation of a positive atmosphere is part of cognitive congruence not of social congruence. | - Biggest sample in all quantitative investigations of p2p-learning - The instrument has been validated across fields of human medicine and years of study - The sample is limited to the Tübingen organizational and curriculum context. |
| **Loda et al. [34]**  *University of Tübingen, Germany*  *Online- und Face-to-Face-Communication class „iTüpferl“* | No information whether same-year or cross-year teaching  **Exploring social and cognitive congruence as the decisive factor making p2p-learning effective**  *Cognitive Congruence*   - Similar language use - Tutor prefers informal contact - No hesitation in case of ambiguities - Stress-free and relaxed learning atmosphere - Comprehensible and informal communication - Creation of an open and nonjudgmental learning environment   *Social congruence*   - time to answer student questions - support, when students face difficulties with the subject matter - empathy by responding to my expectations and needs - interest in students as learners - openness and accessibility to questions and problems - helpful and constructive feedback - has already passed the course | **Social and cognitive congruence**  **Students‘ Learning success** | **Quantitative longitudinal Design**  Standardized testing instrument (Loda et al. 2020)  *Sample:*   - *Student Tutors n=5* - *Students N=128* | Social congruence in the online-setting is significantly higher than in a face-to-face-setting  The data do not show an empirical relation between cognitive congruence and learning success. | - Use of a standardized and validated test instrument for social and cognitive instrument - The need for comparable data has been made a longitudinal virtue. - Tutors take part in a tutor training, that has not been described in the article. - Very small sample of n=5 tutors does not allow to generalize the findings of the study. |
| **Milburn et al. [31]**  *New York, USA*  *4th year students*  *4-week student as teacher elective* | Cross-year teaching  *didactic sessions on topics such as the creation of conducive learning environments, attributes of exceptional teachers, and evidence-based methods of precepting.* |  | **Single case study**  *N=5* | Boost for development as teachers by combining foundational learning with practical experiences.  Mentoring relationships with faculty staff were inspiring  impactful: 94% of leaners agreed with the statement that it was ‘beneficial having fourth-year medical students teaching junior colleagues | Student driven  Experience based |
| **Olthuis et al. [36]**  *Nijmwegen, Netherlands* | Cross-year teaching  **Exploring (learning) experiences of students who collaborated in the organizing committee of a specific a large-scale educational event for fellow students**  *Learning objectives*  *1. organize a large-scale educational event in a team.*  *2. fulfill a specific role in the team and take final responsibility.*  *3. insights in additional matters such as budget / finances, (fire) safety, logistic support and public relations.*  *4. manage peers and students.*  *5. clear and concise communication with all parties involved and establishment of contacts with external partners*  *6. systematic work.*  *7. promote good cooperation within the team* | **Experience/**  **experiential learning**  **ladder of student participation in curriculum design** | **Qualitative exploration design**  focus groups  *sample:*  *longitudinal*  *3 focus groups encompassing organizing teams of 2015/16, 2016/17 and 2017/18* | 1. *Collaboration*: setting up a reliable relation with peers, faculty members and external cooperation partners 2. *Planning and division of labor:* create a realistic planning, prepare for meetings, and work with an agenda, assignment of tasks within the team 3. *Freedom implies responsibility:* participants experienced degrees of freedom and at the same time sometimes stressful responsibility to organize an event 4. *Personal development:* soft skill development outside the medical standard curriculum | - Open approach and innovative approach towards informal learning processes. - The findings do refer to a specific large scale educational event (about 1000 participants). - recall bias may have occurred - not all students participated. Selection bias may have occurred. - group dynamic within a focus group interview may have inhibited critical evaluation of each other. |
| **Ringel et al. [35]**  *University of Heidelberg, Germany*  *Institute of General Internal and Psychometric Medicine* | no information whether cross-year or same-year teaching  **development and implementation of a training course for student tutors**   - *medical content related learning goals* - *Leading small groups* - *Conducting of medical exercises* - *Respectful behavior towards difficult participants* |  | **Single case study**  quantitative questionnaires with open questions focusing on participant satisfaction and estimation of own learning effects  t1, t2  *Sample:*  *N=10 student tutors, who already passed the Basic Heidelberg Tutor Training*  *N=58 students* | The Communication-Trainings-program seems to be effective.  Especially t2 shows significant learning success.  Training amount of all in all 120h is legitimized due to positive learning effects. | - The study consists in a big-sized snapshot limited to Heidelberg University. - The sample size is small. There is no control-group. |
| **Stenberg et al 2021 [37]**  *International*  *Students assessing students in higher education of human medicine, dentistry, nursing, radiology, midwifery, physical or occupational therapy* | no information whether cross-year or same-year teaching  **Scoping review about formative assessment in higher healthcare education**  *Feedback as an important p2p-teacher skill* |  | **Scoping review of scholarly articles and grey literature**  *N =18 articles* | The most frequent setting for formative peer assessment activities is within clinical skill­training courses involving intraprofessional peers.  The common rationale for using formative peer assessment is to support students, usually explained by the inherent learning of the feedback process and to prepare students for professional behavior and provide them with the skills required in the healthcare professions.  Feedback has positive effects on learning.  Feedback needs to be trained. | *Focus on higher healthcare education* |
| **Yeung et al. [32]**  *Toronto,*  *Canada*  *2^nd^ year students*  *7month program*  *Many participants had prior experience* | no information whether cross-year or same-year teaching  *‘Orienting the Learner’*  *‘Principles of Adult Learning’*  *‘Small Group Teaching’*  *Presentation*  *Medical education theories* | **Zone of proximal development (Vygotsky)** | **Single case study**  Mixed method &  Pre-/post-design  + group discussion *N=18* | Very effective training  Respondents report an improvement of their own learning strategies | Limitation:  Individuals with little teaching experience may underestimate their self-perceived teaching skills while those with extensive teaching experience may overestimate their abilities. |
